# Postgastrectomy follow-up in the West: evidence base, guidelines, and daily practice

**DOI:** 10.1007/s10120-016-0654-9

**Published:** 2016-10-07

**Authors:** Magnus Nilsson

**Affiliations:** 1 Division of Surgery, Department of Clinical Science, Intervention and Technology, Karolinska Institutet, Karolinska University Hospital, Kirurggatan 53, 141 86 Stockholm, Sweden; 2 Center for Digestive Diseases, Karolinska University Hospital, Kirurggatan 53, 141 86 Stockholm, Sweden

**Keywords:** Follow-up, Gastric cancer, Gastrectomy, Recurrence surveillance

## Abstract

Follow-up after gastrectomy for gastric cancer has several purposes, including management of side effects of surgery, oncological recurrence surveillance, psychological support, and data collection for research. How follow-up after gastrectomy, and especially recurrence surveillance, is performed differs immensely between different Western countries, despite guidelines from Western oncological organizations quite unanimously advocating symptom-driven surveillance, without scheduled cross-sectional imaging, endoscopies, or analysis of tumor markers. Given a complete lack of randomized data, the available body of observational data does not support intensive routine surveillance for recurrent disease. Moreover, studies of other cancers have shown a negative emotional impact of routine surveillance. There is an apparent need for randomized controlled trials to address the issue of optimized strategies for postgastrectomy recurrence surveillance.

## Introduction

In recent years a number of pivotal randomized controlled trials covering different aspects of gastric cancer management have been performed [1–6], substantially improving the evidence platform on which treatment decisions are made and paving the way for increased standardization of stage-specific management. Despite these advances, there is no international consensus on the best strategy for follow-up after curatively intended gastrectomy for gastric cancer. A recent publication showed that even within the relatively homogeneous group of countries in western and central Europe, national guidelines and practice differ immensely regarding postgastrectomy recurrence surveillance [7].

Follow-up after gastrectomy for gastric cancer has several components that meet different objectives. The first and most immediate reason to follow up patients after gastrectomy is the management of side effects caused by surgery, many of which are associated with eating and nutrition, including malabsorption, weight loss, and subjective alimentary discomfort [8]. Another obvious objective of follow-up after gastrectomy is cancer recurrence surveillance. This is a crucial aspect of follow-up for most cancers, and for some, such as colorectal and breast cancers, there is firm evidence of survival benefit from randomized controlled trials, supporting this practice [9–11].

The design of optimal follow-up programs after gastrectomy for gastric cancer is a complex task, especially balancing the potential benefits and drawbacks of rigorous recurrence surveillance in the context of a total lack of randomized trials addressing this issue with specific regard to gastric cancer. The pivotal question is whether a potential survival benefit of rigorous surveillance outweighs the monetary costs and possible psychological burden of recurrent anxiety caused by frequent surveillance investigations.

## Follow-up components and objectives

Follow-up after surgery for gastric cancer can be categorized by four main objectives: management of side effects after surgery, cancer recurrence surveillance, psychological support, and data collection for treatment evaluation and research.

### Management of complications and postgastrectomy syndrome

All types of gastrectomy for gastric cancer have short-term and long-term effects on gastrointestinal and metabolic function. Some of the short-term problems that patients experience after gastrectomy are related to complications of surgery such as leaks from anastomoses or the duodenal stump or a number of other complications (e.g., ones of pulmonary or cardiovascular origin). However, even in a perfectly uneventful postoperative course, patients experience a number of side effects.

The commonest side effects, often referred to as postgastrectomy syndrome, are related to eating and gastrointestinal tract function and affect virtually all patients to some extent in the first few months after surgery [12, 13]. These side effects include early postprandial satiety, loss of appetite, alteration of taste, nausea/vomiting, and diarrhea. In addition to these expected symptoms, which usually become less apparent with time, there are a number of more specific postgastrectomy symptom complexes, such as dumping syndrome and afferent and efferent loop syndromes, the frequency of which depend on the extent of gastric resection and the type of reconstruction [14].

Important long-term complications following gastrectomy are risks of anemia, caused by deficiencies of iron or vitamin B_12_, and osteoporosis due to malabsorption of vitamin D and calcium. Thus, most surveillance programs include monitoring and supplementation of iron, vitamin B_12_, either orally or parenterally [15], and vitamin D as well as calcium [16].

Most patients experience weight loss after gastrectomy, which is most pronounced in the early phase and subsequently usually stabilizes within the first 2 years after surgery [17]. One of the reasons for the weight loss is probably the discomfort related to eating described above, but it is also to some extent explained by malabsorption [8, 17–19].

### Recurrence surveillance

The surveillance for recurrence of gastric cancer after curative-intent gastrectomy aims at the detection of local recurrence, either in the surgical resection line or in the regional lymph nodes, as well as the detection of distant metastases. There are a number of different ways that recurrence surveillance programs can be designed with different investigational modalities such as cross-sectional imaging with computerized tomography (CT), with or without positron emission tomography, magnetic resonance imaging, ultrasonography, endoscopy, or tumor markers (TM) in blood samples. However, the main line of division is between surveillance programs that actively seek asymptomatic recurrent disease with regular examination with combinations of imaging, endoscopy, and tumor markers and those follow-up programs that offer clinical assessment only at office visits, with targeted investigation only at the occurrence of symptoms or other reasons to suspect recurrence [8].

### Psychological support

A crucial aspect of follow-up after gastric cancer surgery is to provide psychological support and reassurance to the patient and the surrounding family. This is a complex task given the severity of the disease, with a very high recurrence risk and the extremely poor prognosis in the event of recurrence [20–22], with palliative therapy being the only option in the vast majority of patients in whom recurrence is diagnosed. An important question to address in this context is which of the two main surveillance options, regular scheduled investigation aiming at detection of a presymptomatic phase of cancer recurrence, or symptom-driven investigation only, is most beneficial for patients from a specific psychological and broader quality-of-life perspective.

### Data collection for treatment evaluation and research

The systematic gathering and evaluation of data on patients with gastric cancer is of obvious and major importance. Structured follow-up programs after treatment of gastric cancer, not only in the context of prospective clinical trials but also in the daily clinical practice at every hospital and involving every patient in whom gastric cancer has been diagnosed, are important to meet this objective. In many Western countries, such as the UK, Sweden, Denmark, Germany, and the Netherlands, there are national registries or mandatory national audits, with registration of at least all patients undergoing surgery, but in some countries also including nonsurgically treated patients with gastric cancer [23, 24].

## Oncological recurrence surveillance in different Western countries

In a recently published description of clinical pathways for gastric and esophageal adenocarcinoma in ten European countries, it was clearly shown that the pathways used for oncological recurrence surveillance differed immensely, even between these otherwise quite similar western and central European countries [7]. Some countries included in the survey such as Denmark, the Netherlands, Ireland, Poland, Sweden, and the UK based their oncological surveillance mainly on clinical assessment at regular office visits, without scheduled cross-sectional imaging or analysis of tumor markers, performing these or other sophisticated investigations only in the event of clinical suspicion of recurrence. Other countries, including France, Germany, Italy, and Spain, had more elaborate programs with actual surveillance for nonsymptomatic recurrence, including scheduled regular cross-sectional imaging with CT and the examination of tumor markers in blood samples. In Poland the clinical evaluations at office visits were supplemented with ultrasonography, and in some of these countries endoscopy was also used for surveillance of local recurrence or new primary tumors in patients with a remnant stomach [7].

Likewise, the practice of recurrence surveillance after gastrectomy for gastric cancer is likely to differ considerably in other Western countries and regions (e.g., in North America, Australia, and New Zealand). However, to my knowledge there are no publications available documenting the postgastrectomy practices in these countries and regions.

## Western guidelines and consensus documents

There are a number of guidelines and consensus documents issued by national and international professional organizations addressing the issue of postgastrectomy follow-up for gastric cancer patients. In some contrast to the differences discussed earlier regarding follow-up practices in different Western countries, Western guidelines from health-profession organizations are quite unanimous in advocating follow-up with symptom-driven recurrence investigations only. These guidelines include the joint ones from the European Society for Medical Oncology, the European Society of Surgical Oncology, and the European Society of Radiotherapy and Oncology from 2013 [25], the joint ones from the Association of Upper Gastrointestinal Surgeons of Great Britain and Ireland, the British Association of Gastroenterology, and the British Association of Surgical Oncology from 2011 [26], and the guidelines from the US National Comprehensive Cancer Network from 2013 [27] In a consensus document, based on the Charter Scaligero Consensus Conference in Verona in 2013, 48 gastric cancer experts from around the world concluded that follow-up after gastrectomy for cancer should be tailored to the stage of the disease, mainly based on cross-sectional imaging, and should be discontinued after 5 years [28]. It is notable that this is the only published major consensus guideline, with a predominant influence of Western experts, advocating regular cross-sectional imaging.

## Is there evidence of an oncological benefit of postgastrectomy surveillance?

For several other cancers, among them notably colorectal and breast cancer, there are a number of randomized clinical trials that clearly show that intensive surveillance for asymptomatic recurrence increases overall survival compared with symptom-driven recurrence investigation only [9–11]. Characteristic for these cancers, however, and in some contrast to the situation regarding gastric cancer, there is a proven strong survival benefit from intense treatment of recurrent distant metastatic disease, including the quite extensive use of therapy guided by molecular profiling and the proven benefit of liver and lung resections for the removal of metastases [9]. Several series have been published reporting long-term survival in selected patients after resection of metachronous gastric cancer liver metastases. However, these series represent highly selected patients, and there are no data from randomized trials. For gastric cancer, there is clear evidence from randomized clinical trials showing a survival benefit of palliative chemotherapy compared with best supportive care only [29–31], although these effects are modest compared with those for the above-mentioned cancer forms. Hence, given that there is a survival benefit from palliative chemotherapy, there is a reasonable rationale for active surveillance for recurrence assuming that early detection may facilitate chemotherapy as a higher proportion of patients are likely to have a high performance status and be able to tolerate treatment, thus possibly enhancing treatment results. On the other hand, there is likewise a risk that more patients may be treated with more side effects and poorer quality of life, without a significant advantage in terms of increased survival. Let us scrutinize the available evidence.

Unfortunately, there are no published or ongoing randomized trials comparing intensive surveillance for nonsymptomatic recurrence with symptom-driven follow-up for gastric cancer. Thus, all the available evidence addressing this important clinical question is observational in nature. See Table 1 for an overview of studies addressing survival benefit of recurrence surveillance.

**Table 1:** Observational studies addressing gastric cancer recurrence surveillance after gastrectomy

Authors	Publication year	Variables compared	Main outcomes	Results
Bohner et al. [33]	2000	Symptomatic vs asymptomatic recurrence	PRS	Longer PRS in asymptomatic group
Kodera et al. [34]	2003	Symptomatic vs asymptomatic recurrence	PRS, OS	Longer PRS in asymptomatic group. No difference in OS
Bennett et al. [32]	2005	Symptomatic vs asymptomatic recurrence	PRS	Longer PRS in asymptomatic group
Tan and So [35]	2007	Intensive vs less intensive surveillance	Time to recurrence	Shorter time to recurrence for intensive surveillance group, no difference in OS
Eom et al. [36]	2011	Recurrence detected by surveillance vs by symptoms	OS	No difference
Bilici et al. [37]	2013	Symptomatic vs asymptomatic recurrence	DFS, PRS, OS	Longer DFS, PRS, and OS in asymptomatic group
Lee et al. [38]	2014	Symptomatic vs asymptomatic recurrence	DFS, PRS, OS	Longer DFS, PRS, and OS in asymptomatic group
Peixoto et al. [39]	2014	Intensive vs symptom-driven surveillance	OS	No difference
Park et al. [40]	2016	Intensive vs less intensive surveillance	OS	No difference

*DFS* disease-free survival, *OS* overall survival, *PRS* postrecurrence survival

In 2012 Cardoso et al. [22] published a systematic review summarizing the studies available at that time. All five studies selected to relevantly address the issue were retrospective observational studies, and the authors’ conclusion was that there was no evidence to suggest that surveillance after gastrectomy for gastric cancer had any survival benefit. Three of the studies [32–34] simply compared patients with symptomatic recurrences with those with asymptomatic recurrences. They all showed significantly increased postrecurrence survival in patients with asymptomatically detected recurrences, a finding which may very well be entirely explained by lead-time bias. The only one of these three studies that reported overall survival did not observe any difference between symptomatically and asymptomatically detected recurrences [34]. The study by Tan and So [35] compared an intensive follow-up regimen that included twice annual clinical examination, CT, and tumor marker assessment with a regimen with maximal once annual investigation. They found that the intensive surveillance significantly shortened the time to detection of recurrence from a mean of 19.2 months in the low-intensity surveillance group to 11.5 months in the intensive surveillance group (*P* = 0.02), while not significantly affecting overall survival after surgery.

In recent years a few more observational studies addressing the issue of the oncological benefit of intensive surveillance have been published [36–40], Bilici et al. [37] published a retrospective series in 2013, where they, like most previous authors, compared symptomatic recurrences with asymptomatic recurrences and showed significantly longer overall survival among patients with asymptomatic recurrence, in addition to the expected longer postrecurrence survival. There was a slightly longer disease-free survival, reflecting the time to detection of recurrence, in the asymptomatic group. Bilici et al. suggest that these findings may be explained by symptomatic recurrence perhaps being a marker of biological aggressiveness of the cancer. Likewise, Lee et al. [41] found that both postrecurrence survival and overall survival were longer in patients with asymptomatic recurrences, whereas the time to recurrence did not differ, indicating that the increased survival in the asymptomatic group may indeed be due to selection of patients with less aggressive disease rather than to an intervention benefit of surveillance [41].

Recent studies addressing surveillance regimens of different intensity have failed to show any difference in overall survival between these [39, 40]. In a Canadian cohort study based on prospectively collected data, Peixoto et al. [39] studied patients operated on for gastroesophageal cancer with curative intent and followed up with regimens of different diagnostic intensity. They concluded that after multivariably adjusted analyses, there was no difference in overall survival in a comparison of symptom-driven follow-up with more vigorous surveillance that included regular imaging. Lastly, Park et al. [40], in a study from Korea, compared surveillance programs with CT examinations of different frequency after gastrectomy for gastric cancer, ranging from CT every 3 months to every 6–12 months, but did observe any difference in overall survival between these groups.

The role of endoscopy in surveillance for cancer recurrence after R0 gastrectomy is very limited [8, 38, 43]. On the other hand, endoscopy does have a significant role in searching for second primary gastric cancers after nontotal gastrectomy [42–44]. Patients who undergo resection for gastric cancer have a significantly increased risk of new primary gastric cancers, and the prognosis in these patients is excellent if detection is early, but poor if a second primary tumor is detected at a later stage (T2 or higher) [44–46].

## Psychological aspects

There are no published studies regarding the impact of gastric cancer recurrence surveillance with regard to its psychological effects or effects on health-related quality of life. There is, however, a large body of literature concerning the psychological impact of recurrence surveillance in other cancer forms, especially breast, prostate, and colorectal cancer [47–49]. It is evident from these studies that many patients experience severe anxiety related to testing for recurrence or disease progression.

An important difference between gastric cancer and breast, prostate, and colorectal cancers is that the prognosis, if a recurrence is diagnosed, is relatively good compared with the generally very short life expectancy of patients with recurrent gastric cancer. Adult aggressive lymphoma is a malignant disease with a high degree of prognostic resemblance to gastric cancer. Thompson et al. [50] reported on the psychological impact of recurrence surveillance CT scans after curative-intent treatment of patients with this disease. Using mixed qualitative interview and quantitative techniques, they observed that patients experienced significant anxiety related to the surveillance CT scans, and they concluded that “it is possible that the harm of routine surveillance scans for survivors of aggressive lymphoma may outweigh the value, given the lack of randomized data on the effectiveness of the current practice standards, false-positive findings, high cost, radiation exposure, and negative emotional impact on patients.”

## Conclusions

Follow-up after gastrectomy for gastric cancer has several purposes, including management of side effects after surgery, oncological recurrence surveillance, psychological support, and data collection for research. Despite the fact that guidelines from Western health professional organizations are quite unanimous in recommending symptom-driven recurrence surveillance only, practice in Western countries differs, and often includes intensive surveillance with CT and analysis of tumor markers. There are no data available from randomized controlled trials addressing how recurrence surveillance after gastrectomy for gastric cancer should best be performed. However, the available observational evidence does not support routine surveillance for asymptomatic cancer recurrence. In addition, there is some evidence from other cancers indicating that intensive routine surveillance may have a negative emotional impact on patients. In conclusion, there is a strong need for randomized clinical trials addressing postgastrectomy surveillance intensity with regard to survival, psychological impact, and health-related quality of life.
